# Long-Term Effects of Fertility Treatments on Maternal Health

**DOI:** 10.7759/cureus.72464

**Published:** 2024-10-27

**Authors:** Faiza Rahman, Nabila Shams, Laraib Fatima, Laiba Asad, Kashmal Khattak, Ayesha Khan, Hina Khan

**Affiliations:** 1 Department of Obstetrics and Gynecology, Rehman Medical Institute, Peshawar, PAK; 2 Department of Obstetrics and Gynecology, Hayatabad Medical Complex, Peshawar, PAK; 3 Department of Obstetrics and Gynecology, Pak International Medical College, Peshawar, PAK

**Keywords:** assisted reproductive technologies, cancer, cardiovascular disease, chronic conditions, fertility treatment, hormonal therapies, ivf, long-term health effects, maternal health, metabolic disorders

## Abstract

Background

The long-term health effects of assisted reproductive technologies, including in vitro fertilization (IVF) and hormonal therapies, remain poorly understood. This highlights the need to investigate potential chronic conditions that may arise following treatment.

Objective

The objective of this study was to investigate the long-term effects of fertility treatments on maternal health, with a focus on the incidence of chronic conditions and overall health outcomes in women who have undergone such treatments.

Methodology

This cross-sectional study included 180 women, aged 20-45, who had received fertility treatments and successfully delivered a live child. The research was conducted at Rehman Medical Institute and Hayatabad Medical Complex in Peshawar, Pakistan, from January to December 2023. Data were collected through medical record reviews and structured interviews to assess long-term health outcomes, including cancer, metabolic disorders, and cardiovascular diseases. Descriptive statistics and logistic regression were applied using IBM SPSS Statistics for Windows, Version 25.0 (Released 2017; IBM Corp., Armonk, NY, USA), with a significance threshold set at p < 0.05.

Results

The study included 180 participants with an average age of 34.5 years. Among them, 73 (40.56%) underwent hormone therapy, 81 (45.00%) used IVF, and 26 (14.44%) used intracytoplasmic sperm injection. All participants delivered live births, with 21 (11.67%) experiencing premature deliveries and 33 (18.33%) having multiple pregnancies. Key findings showed that nine (5.00%) developed cancer, 31 (17.22%) had metabolic disorders, and 24 (13.33%) developed cardiovascular disease. Overall, 146 (81.11%) participants reported excellent long-term health, while 34 (18.89%) had new diagnoses, including 12 (6.67%) with cardiovascular disease, 18 (10.00%) with metabolic disorders, and four (2.22%) with cancer. Additionally, 36 (20.00%) had preeclampsia, 21 (12.00%) experienced OHSS, and 12 (7.00%) developed gestational diabetes.

Conclusions

Despite most participants reporting excellent overall health, the study underscores the need for ongoing monitoring and management, as it reveals significant long-term health risks associated with fertility treatments.

## Introduction

Due to the increasing number of couples facing difficulties conceiving naturally, fertility treatments are becoming more common [1]. These treatments, ranging from modern assisted reproductive technologies (ARTs) such as in vitro fertilization (IVF) to hormone therapies like gonadotropins and other medications that stimulate ovarian function and ovulation, have given hope to many individuals and couples wishing to start a family [2]. The popularity and success of these procedures have led to significant advances in reproductive medicine, enhancing accessibility and success rates [3]. However, the potential long-term health consequences for mothers undergoing these treatments are often overshadowed by the immediate focus on pregnancy outcomes [4].

Given the complex hormonal and physiological changes induced by fertility treatments, maternal health has become a critical area of research [5]. Most reproductive treatments involve hormonal therapies, which are associated with several short-term side effects, including weight gain, mood swings, and ovarian hyperstimulation syndrome (OHSS) [6]. While the acute effects are well-documented and generally manageable, there is growing concern over potential long-term health issues, such as metabolic disorders, increased cancer risk, and cardiovascular complications [7]. These concerns highlight the need for a comprehensive understanding of the long-term impact of fertility treatments on maternal health [8].

Current research predominantly focuses on the safety and efficacy of fertility treatments in achieving successful pregnancies, with less attention given to the long-term health effects on mothers [9]. Studies have indicated that health trajectories in women using ART may differ from those in women conceiving naturally [10]. For instance, some evidence suggests a higher prevalence of gestational diabetes, preeclampsia, and other pregnancy-related complications among women undergoing ART [11,12]. However, the long-term effects on chronic conditions, such as metabolic syndromes and cardiovascular diseases, remain insufficiently explored, necessitating more in-depth longitudinal studies [13,14].

Despite advances in fertility treatment and short-term success rates, a clear gap exists in research concerning the long-term effects of these therapies on maternal health. Many studies lack comprehensive health assessments and extended follow-up periods, limiting our understanding of the potential risks and outcomes that may arise years after treatment.

Objective

The objective of this study was to investigate the long-term effects of fertility treatments on maternal health, with a focus on the incidence of chronic conditions and overall health outcomes in women who have undergone these treatments.

## Materials and methods

Study design and settings

This cross-sectional study was conducted at Rehman Medical Institute and Hayatabad Medical Complex in Peshawar, Pakistan, over a one-year period from January to December 2023.

Inclusion and exclusion criteria

The study included women aged 20-45 who had received fertility treatments between 2018 and 2022 and successfully conceived a live child. Exclusion criteria included women with prior chronic diseases before undergoing fertility treatments, those who had received repeated reproductive treatments at various institutions, or those who had not provided informed consent.

Sample size

The study involved 180 women who met the inclusion criteria. The sample size was determined based on the availability of eligible volunteers and the scope of the study.

Data collection

Data were collected through two methods: reviewing medical records and conducting structured interviews. Medical records provided details on the type of fertility treatment received, the duration of treatment, and the outcomes of the first pregnancy. Structured interviews were conducted to assess long-term health outcomes, including the prevalence of chronic conditions such as cancer, metabolic disorders, and cardiovascular diseases. Participants were also asked about their general health and any new diagnoses following fertility treatment.

Statistical analysis

Data analysis was conducted using IBM SPSS Statistics for Windows, Version 25.0 (Released 2017; IBM Corp., Armonk, NY, USA). Descriptive statistics, including means, medians, and standard deviations, were calculated for continuous variables. Categorical variables were represented using frequencies and percentages. Logistic regression analysis was employed to identify potential risk factors associated with long-term health outcomes, with a significance threshold set at p < 0.05.

Ethical approval

Ethical approval for the research was granted by the Ethical Review Board of Rehman Medical Institute. All participants provided written informed consent before being included in the study. They were assured of their right to withdraw from the study at any time without facing any repercussions, and measures were taken to ensure the confidentiality and privacy of their data.

## Results

Table 1 presents the demographic profile of the 180 participants in the study. The participants had a mean age of 34.5 years (SD ± 5.6), with the age distribution as follows: 46 participants (25.56%) were aged 20-29 years, 81 participants (45.00%) were aged 30-39 years, and 53 participants (29.44%) were aged 40-45 years. All participants were married. Regarding educational attainment, 89 participants (49.44%) held a bachelor’s degree, 57 participants (31.67%) had a master’s degree, 13 participants (7.22%) possessed a PhD, and 21 participants (11.67%) had completed high school.

**Table 1:** Demographic characteristics of study participants

Characteristic		Number of patients (n)	Percentage (%)
Age (years)	20-29	46	25.56
	30-39	81	45
	40-45	53	29.44
	Mean ± SD	34.5 ± 5.6	
Marital status	Married	180	100
Educational level	High school	21	11.67
	Bachelor’s degree	89	49.44
	Master’s degree	57	31.67
	Doctorate	13	7.22

**Figure 1:**
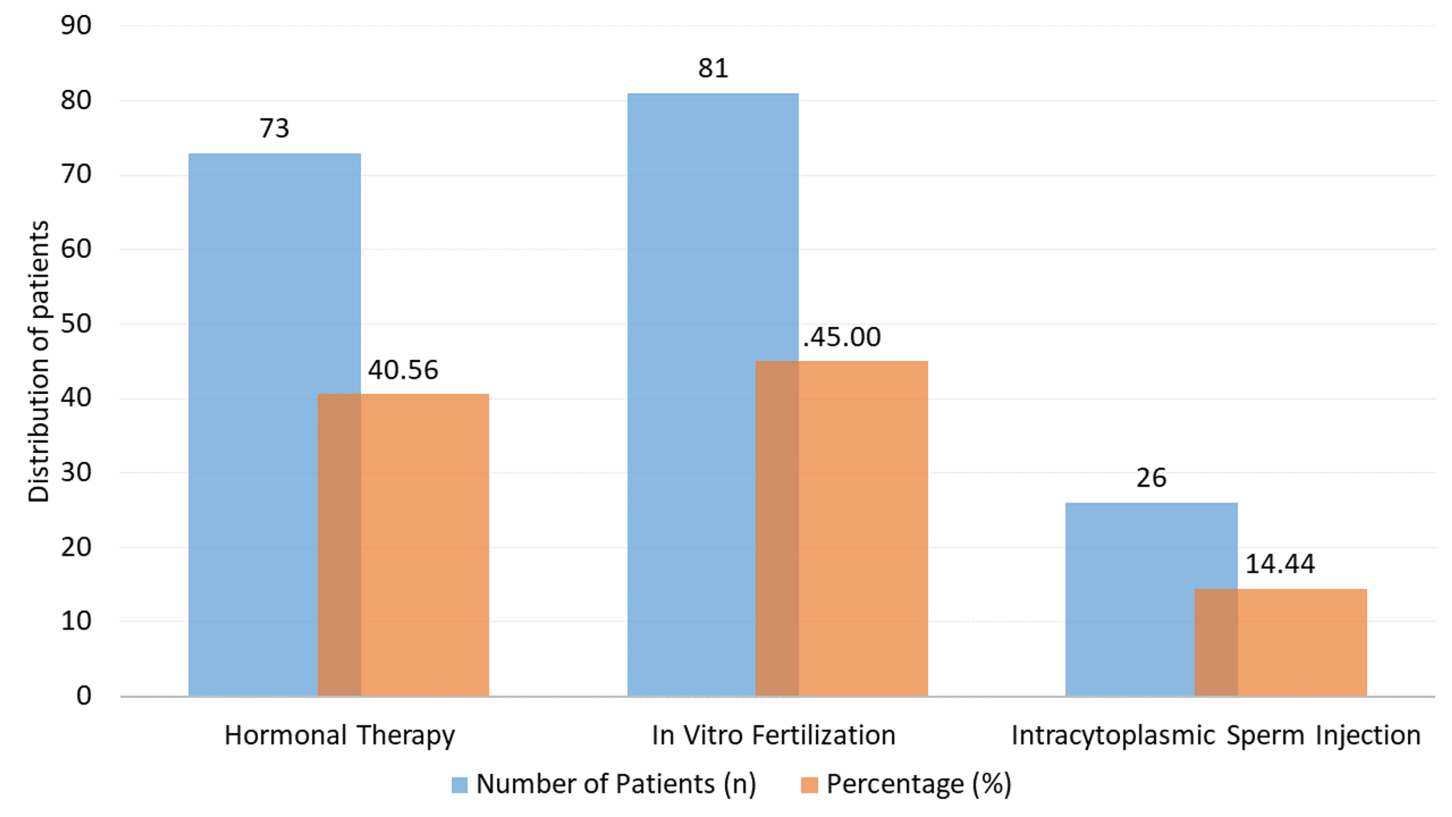
Distribution of fertility treatment types

Figure 1 illustrates the distribution of fertility treatment modalities among the 180 participants. Among the sample, 40.56% (73 patients) underwent hormonal therapy, while IVF was the most commonly used treatment, accounting for 45.00% (81 individuals) of the total. Additionally, 14.44% of the participants (26 individuals) received intracytoplasmic sperm injection (ICSI).

**Figure 2:**
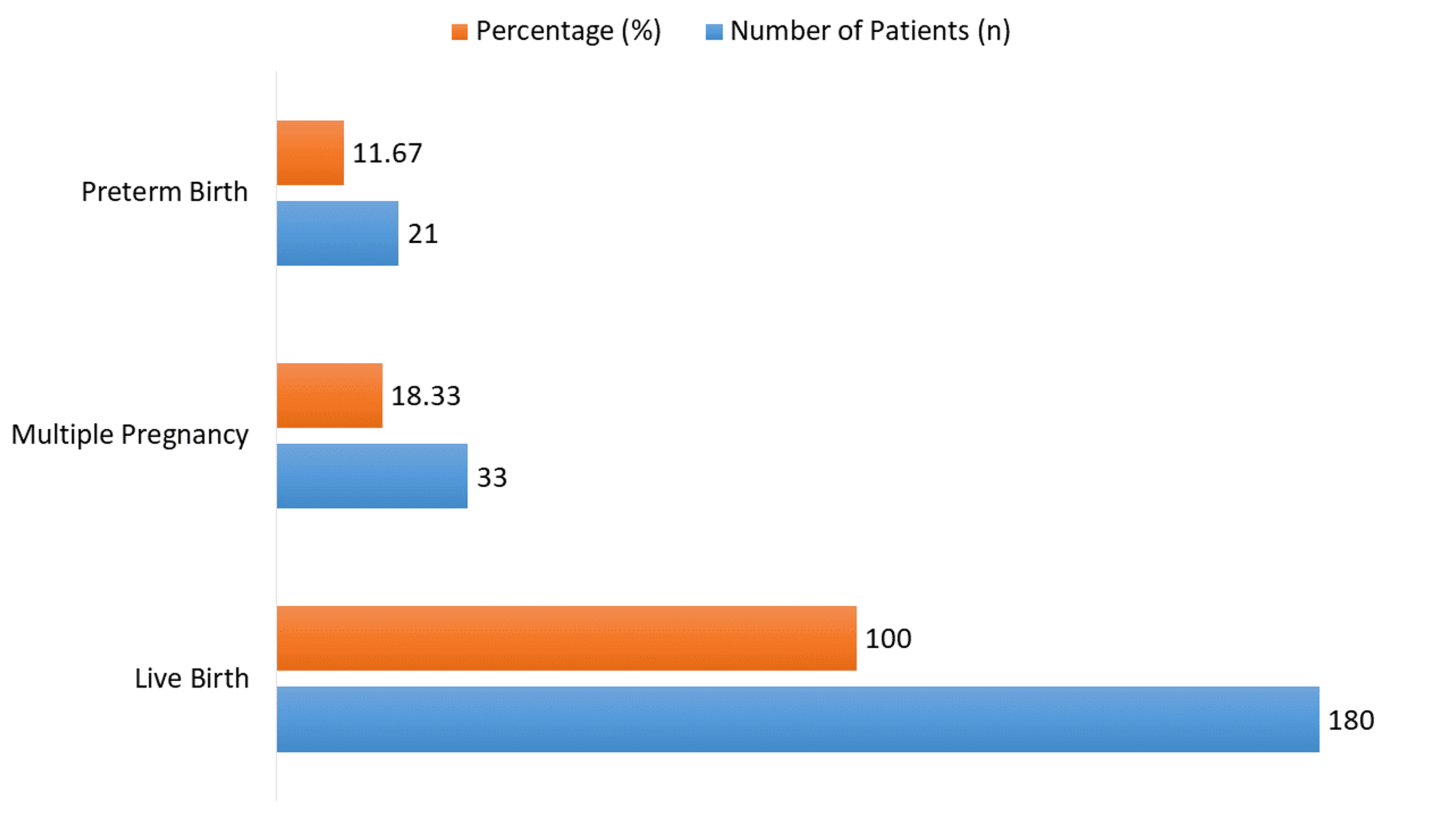
Immediate pregnancy outcomes following fertility treatments

Out of the 180 patients, 33 (18.33%) experienced multiple pregnancies, indicating the presence of more than one fetus, representing 100% of the sample. Notably, all 180 patients achieved a live birth (Figure 2). Additionally, 21 patients (11.67%) delivered before 37 weeks of gestation, which is classified as preterm birth.

Table 2 presents the incidence of chronic diseases among the 180 patients following therapy. Among the sample, 24 individuals (13.33%) reported having cardiovascular disease. Additionally, 31 patients (17.22%) were diagnosed with metabolic disorders. Cancer was reported in nine patients (5.00%), including cases of breast, prostate, and colorectal cancers. Furthermore, 116 individuals (64.44%) reported having no ongoing medical issues.

**Table 2:** Post-treatment incidence of chronic conditions

Condition	Number of patients (n)	Percentage (%)
Cardiovascular disease	24	13.33
Metabolic disorder	31	17.22
Cancer	9	5
No chronic condition	116	64.44

**Figure 3:**
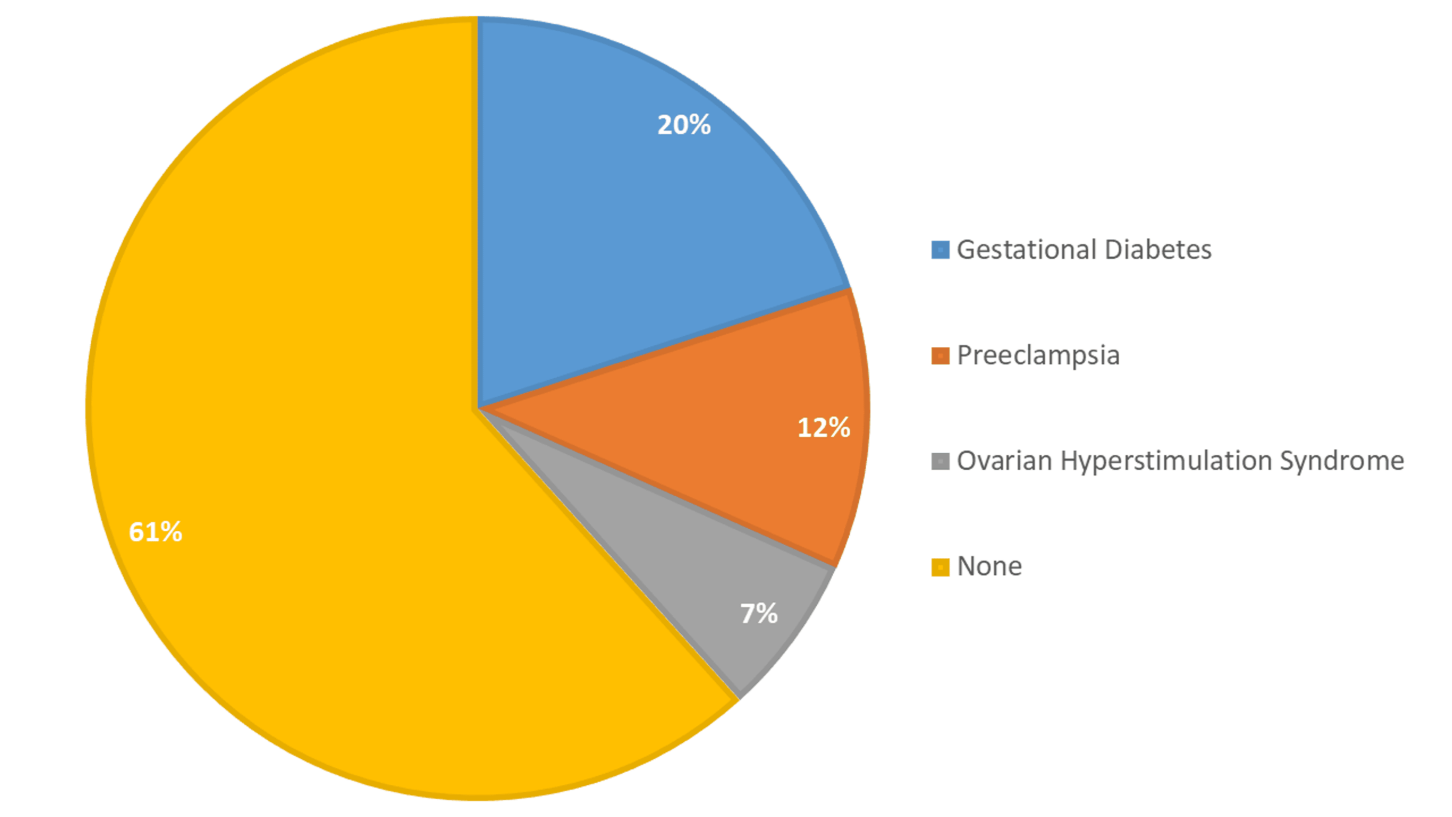
Prevalence of pregnancy-related complications

The prevalence of pregnancy-related complications among the 180 patients is detailed as follows: 36 individuals (20.00%) reported experiencing gestational diabetes. Additionally, 21 patients (12.00%) were diagnosed with preeclampsia, while 12 individuals (7.00%) had OHSS. Furthermore, 111 individuals (61.00%) reported having no complications related to pregnancy (Figure 3).

Table 3 compares the frequency of chronic diseases across different reproductive therapies. Among the 73 individuals receiving hormone therapy, 10 (13.70%) had metabolic disorders, 12 (16.44%) had cardiovascular disease, five (6.85%) had cancer, and 46 (63.01%) reported no chronic illnesses. In the group undergoing IVF (n = 81), 52 patients (64.20%) had no chronic illnesses, while 11 patients (13.58%) had cardiovascular disease, 15 (18.52%) had metabolic disorders, and three (3.70%) had cancer. Among the 26 patients who underwent ICSI, 18 (69.23%) reported no chronic illnesses, while three (11.54%) had cardiovascular disease, four (15.38%) had metabolic disorders, and one (3.85%) had cancer.

**Table 3:** Comparison of chronic conditions by type of fertility treatment

Condition	Hormonal therapy (n = 73)	IVF (n = 81)	ICSI (n = 26)	p-value
Cardiovascular disease	10 (13.70%)	11 (13.58%)	3 (11.54%)	0.918
Metabolic disorder	12 (16.44%)	15 (18.52%)	4 (15.38%)	0.731
Cancer	5 (6.85%)	3 (3.70%)	1 (3.85%)	0.558
No chronic condition	46 (63.01%)	52 (64.20%)	18 (69.23%)	0.408

Table 4 presents the new diagnoses and long-term health status of the 180 patients. A total of 146 patients (81.11%) reported being in overall good health, while 34 patients (18.89%) received a new diagnosis. Among these new diagnoses, 12 patients (6.67%) were identified with metabolic disorders, 18 patients (10.00%) with cardiovascular disease, and four patients (2.22%) with cancer.

**Table 4:** Long-term health status and new diagnoses

Variable		Number of patients (n)	Percentage (%)
Health status	Overall good health	146	81.11
	New diagnosis	34	18.89
Types of new diagnosis	Cardiovascular disease	12	6.67
	Metabolic disorders	18	10
	Cancer	4	2.22

## Discussion

The long-term health outcomes of fertility treatments have garnered increasing attention as more individuals and couples seek these interventions to achieve pregnancy. This research examined several aspects of post-treatment maternal health, yielding both anticipated and unexpected results.

According to our findings, 64.44% of patients reported no chronic diseases, while 13.33% had cardiovascular disease, 17.22% had metabolic disorders, and 5.00% had cancer. These results are consistent with several other studies indicating a higher risk of metabolic and cardiovascular disorders following reproductive treatments [15]. For instance, research by Rossberg et al. [16] found that women who underwent ART had an elevated cardiovascular risk, underscoring the need for long-term monitoring. Additionally, the incidence of metabolic diseases aligns with a study conducted by Udell et al. [15], which revealed that women who underwent reproductive treatments had a higher likelihood of developing metabolic syndrome.

In terms of pregnancy-related complications, 20.00% of patients experienced gestational diabetes, 12.00% had preeclampsia, and 7.00% had OHSS. These rates are somewhat similar to those reported by Wang et al. [11], who noted a significant prevalence of preeclampsia and gestational diabetes among patients receiving antiretroviral therapy. However, the incidence of OHSS in our study was lower than that observed in the study by Sun et al. [17], which could be attributed to variations in patient management techniques or treatment regimens.

When comparing the incidence of cardiovascular disease across different reproductive treatments, it was found to be 13.70% for hormonal therapy, 13.58% for IVF, and 11.54% for ICSI. No significant differences were noted among these groups. This finding is consistent with the research of Dumanski and Ahmed [18], which reported comparable incidences of chronic illnesses across various ART regimens. However, other studies, such as those conducted by Bunting et al. [1], have shown potential variations in long-term outcomes based on specific treatment modalities, despite our data indicating no discernible differences among treatment types.

In terms of long-term health, 81.11% of patients reported being in excellent overall health, while 18.89% received a new diagnosis. Among these new diagnoses, 6.67% were related to cardiovascular disease, 10% to metabolic disorders, and 2.22% to cancer. Although a substantial proportion of patients are in good health, these findings underscore the importance of ongoing surveillance for chronic conditions, as highlighted by Talaulikar and Arulkumaran [19], who emphasized the necessity of long-term health monitoring in ART patients to address emerging health concerns.

In summary, this research contributes to the growing body of knowledge regarding the long-term health effects of reproductive therapies. The observed patterns and comparisons with existing literature, even in the absence of statistically significant differences in certain areas, underscore the need for further investigation and improved patient management strategies to mitigate potential long-term risks associated with reproductive treatments.

## Conclusions

This research underscores the importance of assessing the long-term health effects of reproductive treatments on mothers. While immediate outcomes often receive the most attention, our findings reveal that women who undergo these therapies experience significant rates of cancer, metabolic disorders, and cardiovascular disease. Although a majority of patients maintain excellent health - 64.44% reporting no chronic diseases - our data highlight that a notable minority face long-term health challenges. This emphasizes the necessity for ongoing surveillance and long-term health monitoring for individuals receiving reproductive treatments. Future research should focus on extending follow-up periods and investigating the relationship between specific reproductive treatments and long-term health outcomes to enhance patient care and management strategies.
